# Bimorph material/structure designs for high sensitivity flexible surface acoustic wave temperature sensors

**DOI:** 10.1038/s41598-018-27324-1

**Published:** 2018-06-13

**Authors:** R. Tao, S. A. Hasan, H. Z. Wang, J. Zhou, J. T. Luo, G. McHale, D. Gibson, P. Canyelles-Pericas, M. D. Cooke, D. Wood, Y. Liu, Q. Wu, W. P. Ng, T. Franke, Y. Q. Fu

**Affiliations:** 1 Faculty of Engineering and Environment, Northumbria University, Newcastle upon Tyne, NE1 8ST UK; 2 State Key Laboratory of Electronic Thin Films and Integrated Devices, University of Electronic Science and Technology of China, Chengdu, 610054 P.R. China; 3 College of Intelligent Science and Engineering, National University of Defense Technology, Changsha, HuNan 410073 P.R. China; 4 Shenzhen Key Laboratory of Advanced Thin Films and Applications, College of Physics and Energy, Shenzhen University, Shenzhen, 518060 P.R. China; 5 Institute of Thin Films, Sensors & Imaging, University of the West of Scotland, Scottish Universities Physics Alliance, Paisley, PA1 2BE UK; 6 Department of Engineering, Durham University, South Road, Durham, DH1 3LE UK; 7 Biomedical Engineering, University of Glasgow, Rankine Building, G12 8LT Glasgow, UK

## Abstract

A fundamental challenge for surface acoustic wave (SAW) temperature sensors is the detection of small temperature changes on non-planar, often curved, surfaces. In this work, we present a new design methodology for SAW devices based on flexible substrate and bimorph material/structures, which can maximize the temperature coefficient of frequency (TCF). We performed finite element analysis simulations and obtained theoretical TCF values for SAW sensors made of ZnO thin films (~5 μm thick) coated aluminum (Al) foil and Al plate substrates with thicknesses varied from 1 to 1600 μm. Based on the simulation results, SAW devices with selected Al foil or plate thicknesses were fabricated. The experimentally measured TCF values were in excellent agreements with the simulation results. A normalized wavelength parameter (e.g., the ratio between wavelength and sample thickness, *λ*/*h*) was applied to successfully describe changes in the TCF values, and the TCF readings of the ZnO/Al SAW devices showed dramatic increases when the normalized wavelength *λ*/*h* was larger than 1. Using this design approach, we obtained the highest reported TCF value of −760 ppm/K for a SAW device made of ZnO thin film coated on Al foils (50 μm thick), thereby enabling low cost temperature sensor applications to be realized on flexible substrates.

## Introduction

Temperature monitoring is essential for electrical equipment and mechanical systems; thus various temperature sensors, such as semiconductor oxide sensors, optical fiber sensors and infrared sensors, have been widely used in industry^1–4^. However, current temperature sensing technologies have serious limitations associated with power supply and data transmission. Passive operation and wireless interrogation are often required in many hazardous environments, such as moving machinery, contaminated areas, chemical or vacuum chambers and high voltage plants. In these applications, acoustic waves, especially surface acoustic wave (SAW) based sensors have significant advantages as they provide capabilities of wireless readout, battery-free operation, real-time and remote data communication^5–10^. They also have other merits including high accuracy, low or no maintenance, light weight, reliability and robustness.

In order to use an acoustic wave (for example, SAW) sensor for temperature sensing, one of the key parameters is the temperature coefficient of frequency (TCF), defined as rate of frequency change with temperature relative to a resonant frequency. The TCF values of SAW devices are linked with their thermal stability^9,11^. Most materials have negative TCFs, which means the frequency of the SAW device decreases with an increase in temperature. However, for most sensing applications, such as those for gas, pressure, humidity, chemicals and biosensing, thermal stability is highly desired. Therefore, most researchers use techniques to reduce the TCF values or achieve a temperature compensation during sensing. This can be easily implemented using an additional compensation layer such as silicon dioxide^12–15^ or alumina^16,17^, both of which have positive TCFs. In contrast, for temperature sensing applications, a large absolute value of the TCF with a good linearity is desired. However, so far, there are few reports on how to maximize the TCF values by choosing different materials and/or designing various multilayer structures.

Based on literature, the TCF values of SAW devices made from common piezoelectric materials such as LiNbO_3_, ZnO, AlN, and GaN generally range from −18 ppm/K to −75 ppm/K^10,18–21^. Therefore, in order to increase temperature sensitivity, most researchers increased the resonant frequencies of SAW devices (up to hundreds of MHz and GHz), which will increase the responses for sensing but dramatically increase the fabrication costs and complexity of process/measurement. An alternative approach is to maximize the TCF values of SAW devices by choosing suitable low-cost materials and/or multilayer designs, without the need for significant increase in resonant frequencies.

A further essential challenge in temperature sensing is to detect or monitor changes on a curved or bendable surface, such as those for healthcare applications, and for this purpose, a temperature sensor needs to be mechanically flexible or bendable. High TCF readings have previously been reported for ZnO film based SAW devices fabricated onto polymer (such as polyimide), which are flexible substrates^22–24^. For SAW devices with frequencies of 132 MHz and 427 MHz, TCF values were reported to be −423 ppm/K and −258 ppm/K, respectively^23^, attributed to the large thermal expansion coefficient (TEC) of the polymer substrate. However, there are challenges associated with the ZnO/polymer SAW devices, including significant attenuation and dissipation of acoustic wave energy, poor film crystallinity and poor adhesion of thin films. Al foil or thin Al plates, on the other hand, could be used as an alternative substrate for flexible or bendable SAW devices, reducing the problem of acoustic wave damping and, most importantly, also showing very high TCF values, which were reported by Liu *et al*.^25^. Therefore, it is promising to use Al foils or thin plates to generate high TCF values for flexible temperature sensing applications.

However, a better theoretical understanding of the design mechanisms and experimental studies are urgently required to create structures with optimal TCF values. In this work, we show how ZnO/Al SAW devices with the ZnO films coated onto various Al foils and thin plates (where foil is defined as a layer with a thickness of a few microns to less than 100 microns and a plate defined as a layer with thickness of ~100 microns to about 1 mm) can be appropriately designed to maximise the TCF values. Theoretical analysis and simulations based on finite element analysis (FEA) are presented and the results are verified experimentally. The key objective of the work is to achieve the highest possible TCF readings in a flexible substrate device suitable for temperature sensing.

## Principle and Design Methodology for Achieving High TCFs

The TCF of a layered SAW structure depends on both the temperature variation of acoustic properties and temperature expansion coefficient (TEC) of each material. The theoretical TCF is defined by the following equation^9^:

1 $$TCF=\frac{1}{{f}_{o}}\frac{\partial f}{\partial T}=\frac{1}{{v}_{p}}\frac{\partial {v}_{p}}{\partial T}-\frac{1}{\lambda }\frac{\partial \lambda }{\partial T}=\frac{1}{{v}_{p}}\frac{\partial {v}_{p}}{\partial T}-\alpha ^{\prime} $$

where *f*, *T*, *v*_*p*_, *λ* and *α′* are frequency, temperature, phase velocity, designed wavelength and TEC of the multi-layer structure, respectively. For Al, the TEC value is about 23.6 ppm/K, which is quite large among the most commonly used metals and ceramic materials. Apart from the TEC values, there are two other factors which are critical for the TCF values of the SAW devices, and thus will be discussed as follows.

### Deformation of bi-layer from thermal expansion

The interfaces between the substrate (such as Al foil or thin plate) and ZnO thin film are assumed to be perfectly bonded during heating/cooling from room temperature to 150 °C, where strain due to lattice mismatch is neglected in the analysis. As the temperature increases, the differences in the TECs of ZnO and Al substrate result in the bending of the layered structure. This deformation will also contribute to the strain values due to thermal expansion of two layers, which can be expressed as:

2 $${\varepsilon }_{Al}={\alpha }_{Al}{\rm{\Delta }}T+\frac{{F}_{Al}}{w{h}_{Al}{E}_{Al}}+\frac{{h}_{Al}}{2R}$$

3 $${\varepsilon }_{ZnO}={\alpha }_{ZnO}{\rm{\Delta }}T-\frac{{F}_{ZnO}}{w{h}_{ZnO}{E}_{ZnO}}-\frac{{h}_{ZnO}}{2R}$$

where *α*, *F*, *w*, *h*, and *E* are the TEC, force, width, thickness and Young’s modulus of each layer respectively, whilst 1/*R* is the curvature of the bending structure. We assume that during heating/cooling, there is an equilibrium of moments and no slippage, therefore,

4 $${F}_{Al}={F}_{ZnO}=\frac{2}{{h}_{Al}+{h}_{ZnO}}(\frac{{E}_{Al}{I}_{Al}+{E}_{ZnO}{I}_{ZnO}}{R})$$

5 $${\varepsilon }_{Al}={\varepsilon }_{ZnO}$$

where

6 $$I=\frac{w{h}^{3}}{12}$$

From Eqs (2) and (4)

7 $$\frac{1}{R}=\frac{({\alpha }_{Al}-{\alpha }_{ZnO}){\rm{\Delta }}T}{\frac{{h}_{Al}+{h}_{ZnO}}{2}+\frac{2({E}_{Al}{I}_{Al}+{E}_{ZnO}{I}_{ZnO})}{w({h}_{Al}+{h}_{ZnO})}(\frac{1}{{h}_{Al}{E}_{Al}}+\frac{1}{{h}_{ZnO}{E}_{ZnO}})}$$

By defining a factor *b* where ${h}_{Al}=b\cdot {h}_{ZnO}$, the strain in the ZnO layer is:

8 $${\varepsilon }_{ZnO}={\alpha }_{ZnO}{\rm{\Delta }}T+\frac{({\alpha }_{Al}-{\alpha }_{ZnO}){\rm{\Delta }}T}{\frac{b+1}{2}+\frac{({b}^{3}{E}_{Al}+{E}_{ZnO})}{6(b+1)}(\frac{1}{b{E}_{Al}}+\frac{1}{{E}_{ZnO}})}(\frac{1}{2}+\frac{{b}^{3}{E}_{Al}+{E}_{ZnO}}{6b(b+1){E}_{ZnO}})$$

Therefore, the design wavelength *λ* is no longer a constant during the temperature change, i.e.,

9 $$\lambda ={\lambda }_{0}(1+{\varepsilon }_{ZnO})$$

The TEC of the Al is much larger than that of ZnO, and also ${\varepsilon }_{ZnO}$ is larger than ${\alpha }_{ZnO}{\rm{\Delta }}T$, thus $\alpha ^{\prime}  > {\alpha }_{ZnO}$. As the Al layer expands and deforms more easily than ZnO, it will contribute more to the frequency shift due to the thermal expansion.

### Temperature coefficient of moduli

If the substrate is thick enough compared to the wavelength, Rayleigh based SAWs will be dominant. However, due to thin nature of Al foils and plates, Lamb waves will be generated if the thickness of Al is of similar order to the ZnO film^9^. The phase velocities *v*_*p*_ of the A0 mode wave (*v*_*pA*_) and S0 mode lamb wave (*v*_*pS*_) propagating in a homogeneous and isotropic plate are given by^26^:

10 $${v}_{pA}=\frac{2\pi h}{{\lambda }_{0}}{(\frac{E^{\prime} }{3\rho })}^{1/2}$$

11 $${v}_{pS}=\sqrt{\frac{E^{\prime} }{\rho }}$$

where $E^{\prime} =E/(1-{\upsilon }^{2})$, *ν* is Poisson’s ratio and *ρ* is the density of the composite. For both of these Lamb wave modes, we have,

12 $$\frac{1}{{v}_{p}}\frac{\partial {v}_{p}}{\partial T}=\frac{1}{2}(\frac{1}{E^{\prime} }\frac{\partial E^{\prime} }{\partial T}-\frac{1}{\rho }\frac{\partial \rho }{\partial T})$$

Unlike those of the polymers, the temperature coefficients of density for metals and ceramics are quite small from room temperature up to 100 °C (the range used in the experimental measurement). Therefore, the densities of both ZnO and Al are assumed to be constant values in this study, whereas the moduli of the materials could be slightly changed with temperature, which could be one of the factors for changes of TCF values. There were previous reports that the temperature moduli coefficients (TMCs) of micro-size of Al wires were much larger than that of the bulk Al^27,28^. When the Al foil or plate becomes much thinner, the Al material below each interdigital transducer (IDT) finger would be within micro sizes (both in thickness and in-plane size). Therefore, it is expected that the elastic properties of thin Al foils would contribute significantly to the device’s TCF. Accordingly, we have designed different SAW devices with various Al substrates thicknesses and various wavelengths of IDTs to measure the TCF values.

### Design Implementation and simulation

Finite element method (FEM) based computational modeling of frequency shift as a function of temperature was first conducted using a multi-physics simulation software COMSOL by coupling solid mechanics module and electrostatics module. A 2D layered model composed of one pair of IDTs with periodic boundary conditions was used in the simulation. The simulated layered structure was composed of an Al foil or plate as the substrate, a ZnO thin film and IDTs. The thicknesses of the ZnO thin film and IDTs were fixed as 5 μm and 120 nm, respectively. The designed wavelengths were varied from 32 μm to 400 μm. The Al substrates in the simulations include three types of foils with different thicknesses (i.e. 1 μm, 10 μm and 50 μm) and five types of plates with different thicknesses (i.e. 100 μm, 200 μm, 400 μm, 600 μm and 1.6 mm). During the simulation, the temperature was increased from room temperature to 75 °C. The values of TECs and TMCs for both the Al and ZnO used in the simulation are listed in Table 1.

**Table 1:** Temperature moduli coefficients and thermal expansion coefficients of Al and ZnO used in the simulations.

	Temperature coefficients of elasticity moduli (ppm/K)				Thermal expansion coefficient (ppm/K)
	TCC_11_	TCC_13_	TCC_33_	TCC_44_	*α*
ZnO^30–32^	−112	−161	−123	−70	4.7
	**TMC**_**wire**_		**TMC**_**bulk**_		***α***
Al^27,28^	−1455		−612		23.6

**Figure 1:**
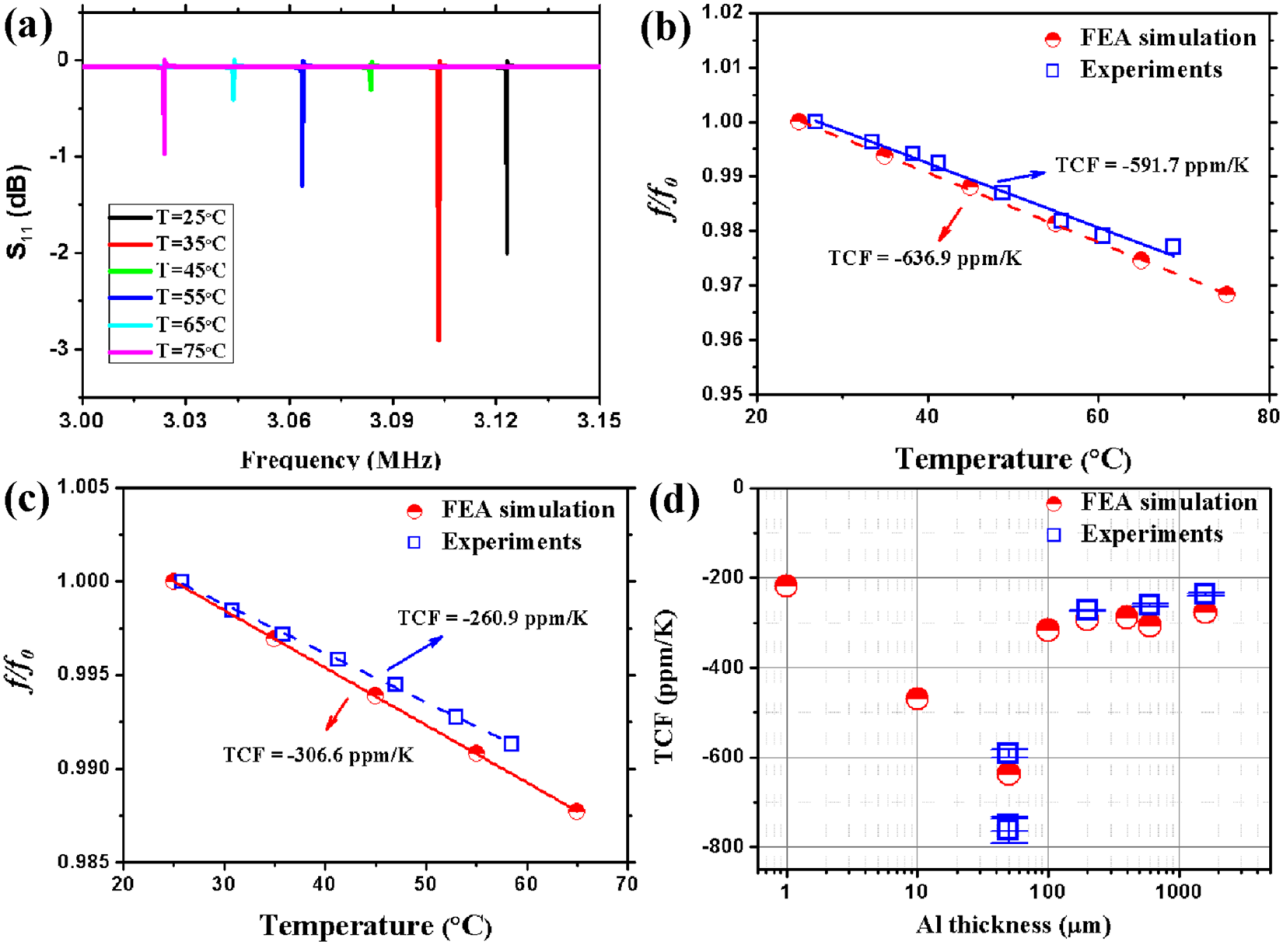
(**a**) Simulated reflection spectra (S_11_) for SAW on Al foil (50 μm thick) with *λ*_*0*_ = 400 μm as temperature increases. The comparison of temperature induced frequency shift between experimental and FEM simulation results for surface acoustic wave devices (**b**) on Al foil (50 μm thick) and (**c**) on Al plate (600 μm thick), all with a designed wavelength *λ*_*0*_ of 400 μm. (**d**) The FEA simulated and experimental TCF results as a function of Al thickness for ZnO film device on Al foils and plates with a designed wavelength *λ*_*0*_ of 400 μm.

As a simulation example, Fig. 1a shows the simulation result of peak shifts of the reflection spectrum (S_11_) for a ZnO SAW device with a wavelength *λ*_0_ = 400 μm on Al foil (50 μm thick) as the temperature is increased. The resonant frequency has a left shift (decreasing trend) with the increase of temperature. The differences between the simulated S11 amplitudes are mainly due to the numerical simulation parameters such as the selections of mesh and frequency sweep resolution. Clearly there is a good linear relationship between frequency shift and temperature changes, and the TCF values can be obtained by calculating the slope of the simulated frequency shift versus temperature (see two examples shown in Fig. 1b,c).

Figure 1d summarizes the simulated TCF results as a function of Al thickness for various ZnO film devices on Al foils and thin plates with a fixed wavelength *λ*_0_ of 400 μm. Due to the micro-scale dimensions (i.e., both thicknesses of the Al foils and the IDT dimensions), we used the modulus data of micro-wires of Al as listed in Table 1^27,28^ for modeling Al foils with thicknesses of 1 μm, 10 μm and 50 μm. From Fig. 1d, the TCF readings are –218.7 ppm/K for 1 μm Al foil, −469.5 ppm/K for 10 μm Al foil and −557.2 ppm/K for 50 μm Al foil. There is an increasing trend with the increase of thickness of Al foils. The TCF values for the 50 μm Al foil sample are quite large, but are supported by our previously reported values of ZnO/Al foil SAW devices^25^.

However, with further increase of Al substrate thickness, the changing trends of the TCF values are different. Figure 1d also shows the numerically simulated TCF results for the acoustic wave devices on Al plates (thicknesses varied from 100 μm to 1.6 mm) with the same designed wavelength, i.e., 400 μm. In this simulation, we used the TMC value of the bulk Al materials as they are thicker plates. The simulated TCF values decrease from −316.6 ppm/K for 100 μm plate to −277.2 ppm/K for 1.6 mm plate, as shown in Fig. 1d.

From the simulation results, we can clearly see that when the Al substrate thickness is increased from 1 micron onwards, the TCF values firstly increase with the Al thickness, but when the Al substrate is gradually changed from a foil to a thin plate as in our definition, the TCF values then decrease with the further increase of the plate thickness. Clearly there is a maximum TCF value at a certain Al layer thickness for the ZnO/Al SAW devices with fixed ZnO film thickness and IDT wavelength. Therefore, based on the guidance of the simulation results, we fabricated various SAW devices with different Al substrate thicknesses, in order to experimentally verify and then achieve the largest TCF value.

## Results and Discussion

**Figure 2:**
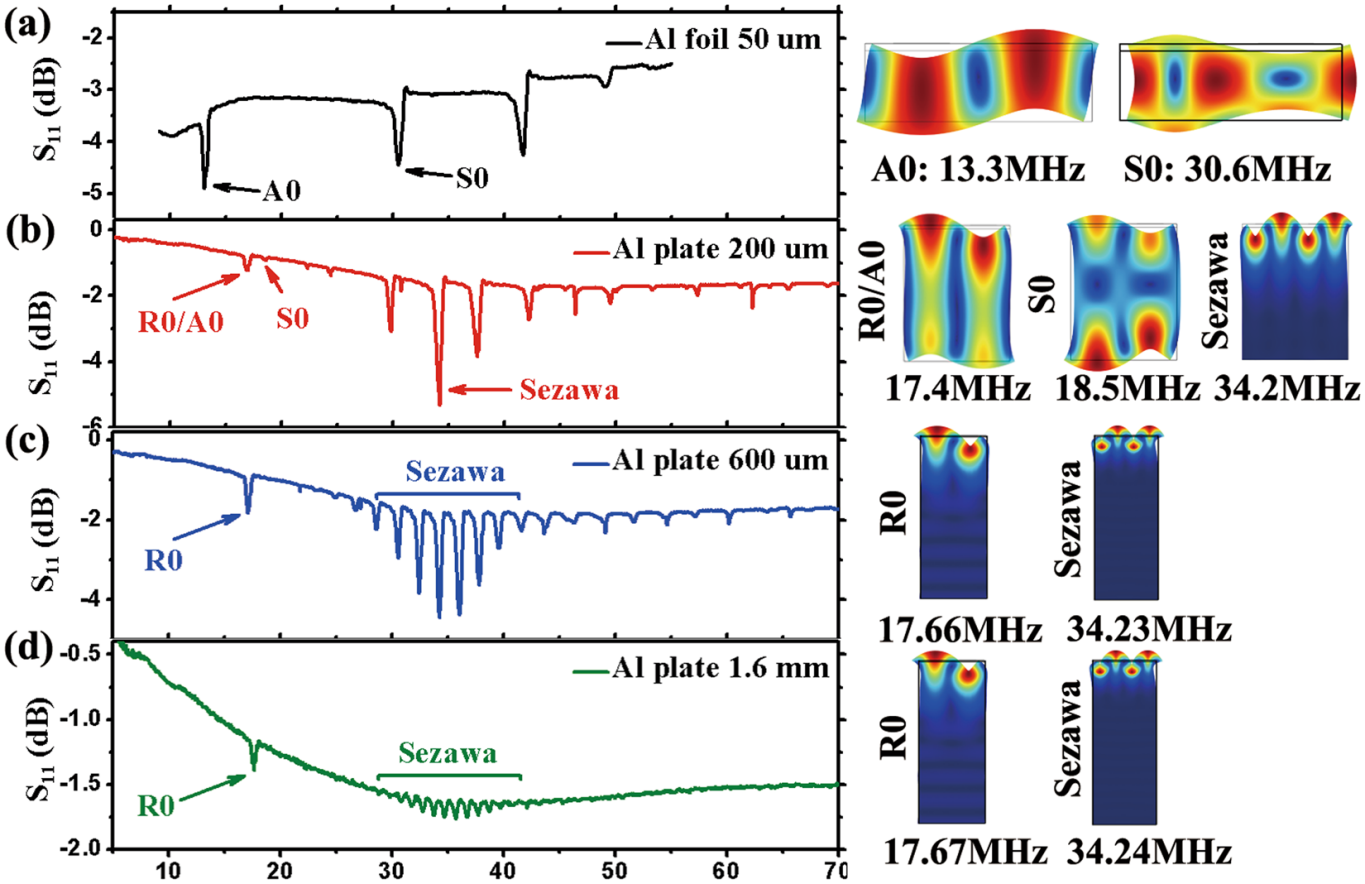
Reflection spectra S_11_ of surface acoustic devices with *λ*_*0*_ = 160 μm integrated on (**a**) Al foil (50 μm thick), (**b**) 200 μm thick Al plate, (**c**) 600 μm thick Al plate and (**d**) 1.6 mm thick Al plate. Wave modes of typical peaks in the spectra were identified based on FEA simulations.

For the ZnO film based acoustic wave devices fabricated on Al foils and thin plates, the wave modes are changed from Lamb waves to Rayleigh waves when the thickness of the Al substrate is increased from 50 μm to 1.6 mm with the same IDT design. For instance, when the IDT designed wavelength *λ*_0_ is 160 μm, the acoustic wave device on Al foil (thickness of 50 μm) excites Lamb waves with both anti-symmetric and symmetric modes (see Fig. 2a); whereas the acoustic wave device on a 200 μm thick Al plate with the same wavelength excites a Rayleigh-Lamb hybrid wave (see Fig. 2b), i.e., the positions of some resonant peaks of these two modes are overlapping. When the Al plates are as thick as 600 μm and 1.6 mm, the surface acoustic wave modes are dominant, i.e., Rayleigh and Sezawa modes (see Fig. 2c,d).

The changes of wave modes can also be understood as their dependence on the ratio between the designed wavelength and the thickness of the device (*λ*_0_*/h*). As the ratio of *λ*_0_*/h* is much larger than 1, the device excites Lamb waves; whereas the device excites Rayleigh and Sezawa modes when the ratio is much smaller than 1. Accordingly, a Rayleigh-Lamb hybrid wave is excited when the ratio is roughly equal to 1 for this study. The wave modes of all the devices were verified from both experimental measurements of reflection spectra S11 and FEA modeling of surface vibration modes; with examples shown in Fig. 2.

When the temperature was increased during the experiment, the resonant frequencies of all acoustic wave modes were shifted to lower values as predicted from the FEM simulations. Figure 1b,c show the experimentally measured frequency results (as a function of temperature) for the ZnO SAW devices on (1) Al foil (50 μm thick); and (2) an Al plate (600 μm thick) with the designed wavelength *λ*_0_ of 400 μm. The experimental data are in good agreements with the simulation results. There are linear relationships between the frequency shift and temperature, and the calculated TCF values are −591.7 and −261.4 ppm/K for these two devices, which are ~6% and ~14% larger compared to the theoretical values from the FEM analysis. Possible reasons for these differences are: (1) absolute accuracy of the chosen values of the TMCs; (2) residual stress in the film accumulated from the fabrication process^29^; and (3) the accuracy of the device’s wavelengths due to the resolution of the lithographic process and/or the rough nature of the Al substrates.

All the TCFs values of the ZnO/Al SAW devices have been measured and the selected readings are shown in Fig. 1d to compare with the simulation results. There is a good agreement between experimental and simulation results, which verify our proposed design methodology. As discussed above, both the designed wavelength and Al thickness influence the variations of the TCF values. Therefore, a normalized wavelength *λ*/*h* was chosen to describe the changes of the TCF values, in which *λ* is the wavelength corresponding to peak frequency and *h* is the total thickness of the layered SAW structure.

**Figure 3:**
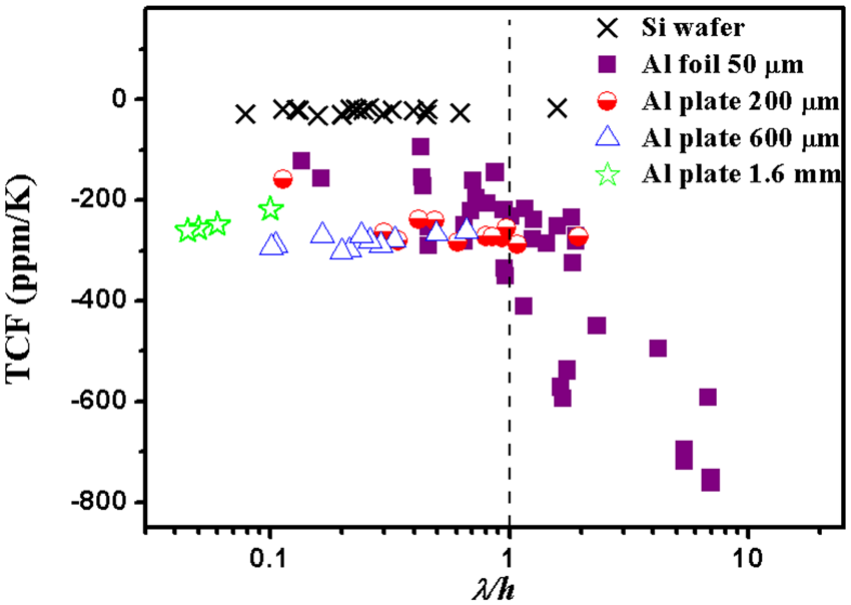
Summary of measured TCF values of ZnO SAW samples based on different Al substrates changing with a normalized wavelength *λ*/*h*. The TCF values of ZnO/Si SAW devices are plotted as a reference.

All the measured TCF values of various samples with different Al substrates as a function of the normalized wavelength *λ*/*h* are summarized in Fig. 3. The TCF values of ZnO based SAWs on Si with different normalized wavelengths (from the literature) are also presented for comparison. The TCF values of ZnO/Si devices are found to be around −20 ppm/K to −50 ppm/K, without significant variations with SAW wavelengths or frequencies^19,30,31^. However, for the SAW devices on different thicknesses of Al substrates with the designed wavelengths varied from 24 μm to 400 μm, their TCF readings are significantly larger than those on the Si wafer, and show dramatic differences as a function of the normalized wavelength of *λ*/*h*.

Based on Fig. 3, when the wavelength is smaller than the ZnO/Al device thickness (i.e., *λ*/*h* < 1), the TCF readings do not show significant variances, and their values are between −200 ppm/K and −300 ppm/K. In this situation, the wave propagation is confined mostly to the top layer of the SAW devices, especially in the ZnO layer. Therefore, the lower and relatively stable TCF readings in these cases can be explained because ZnO has a much smaller value of TCF values. From this trend, if the wavelength is decreased near to the ZnO film thickness, the TCF value of the SAW devices should approach the TCF readings of the ZnO, which is about 50 to 70 ppm/K.

For the acoustic wave devices with a larger value of *λ*_0_ and smaller Al thickness, i.e., with a large value of normalized wavelength *λ*/*h*, the wave velocity could be more influenced by the elastic properties of Al substrates as the waves will propagate in the whole bi-layer structure. Despite the large scattering of the obtained TCF data, there is a general trend that the absolute values of the TCF increase with the normalized wavelength if *λ*/*h* > 1. The highest TCF value obtained in this study is −760 ppm/K at a low frequency of 3 MHz with the design of SAW on Al foil of 50 μm thick with a wavelength of *λ*_0_ = 400 μm. This is the highest value we have seen from all the published TCF data in literature.

However, there are some limitations in experimental work for further increasing normalized wavelength to maximise the TCF values, e.g., application point of view (for larger value of *λ*) and fabrication process (for thicker or thinner values of *h*). A further larger value of *λ* will occupy a large special area on the device surface, and the frequency will be down to a few MHz, which is not good for precision sensing application. Whereas if the Al foil is too thin, the lithography process will become difficult. If the Al substrate is thicker, the film stress generated during the deposition process (due to the ion bombardment during sputtering, and the lattice mismatch and the thermal expansion coefficient mismatch between the ZnO film and Al substrate) causes the increased curling of the ZnO/Al film, which increases the difficulty for lithography process.

**Figure 4:**
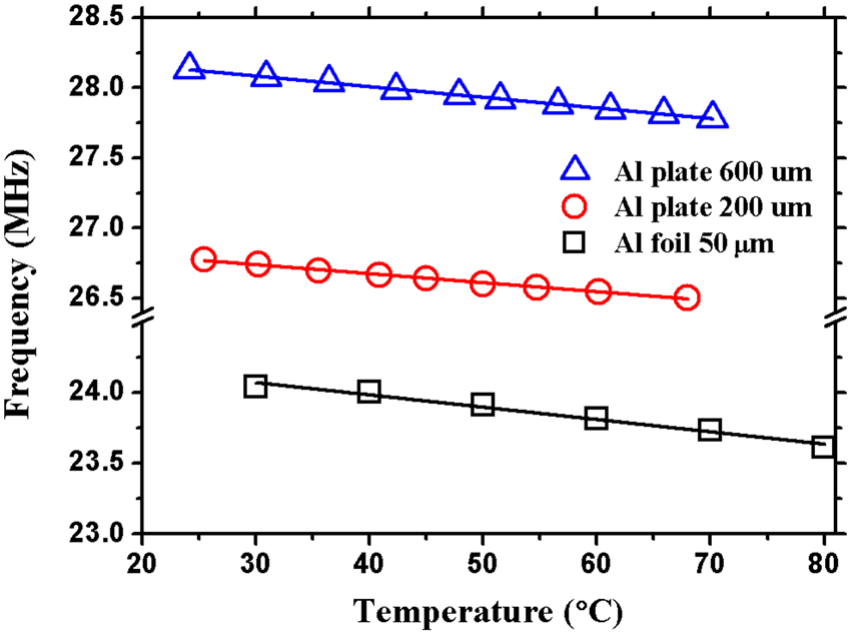
Linear frequency shift with temperature variations of SAWs (λ_0_ = 100 μm) on Al foil (50 μm) and Al plates (200 μm and 600 μm).

Besides the absolute values of the TCF readings, a good linearity is also critical for a precision temperature sensor. The S_11_ frequency signals for SAW devices on all the Al substrates generally show highly linear frequency shifts as a function of temperature. For the same IDT design (λ_0_ = 400 μm), samples (in Fig. 1) on Al foil (50 μm thick) and Al plates (200 μm and 600 μm thick) have the sensitivity readings of 2.06, 1.65 and 1.88 kHz/K, with the corresponding TCF values of −664.6 ppm/K, −270.5 ppm/K and −260.4 ppm/K, respectively. Apart from those shown in Fig. 1, Fig. 4 shows TCF data examples of device with other wavelength values, indicating that the frequency data decrease linearly with the increase of the temperature. For the same IDT design (λ_0_ = 100 μm), samples on Al foil (50 μm thick) and Al plates (200 μm and 600 μm thick) have the sensitivity readings of 8.7, 6.43 and 7.59 kHz/K, with the corresponding TCF values of −362.5 ppm/K, −239.9 ppm/K and −270.1 ppm/K, respectively. The large TCF reading would be a great advantage in practice considering the low cost and easy fabrication of these SAW devices for these low frequencies, while they still have very high sensitivities for temperature sensing. We further verified that the TCF reading generally remained a constant value by testing bent samples on a curved surface, and the temperature sensitivity of the device was not influenced apparently.

**Figure 5:**
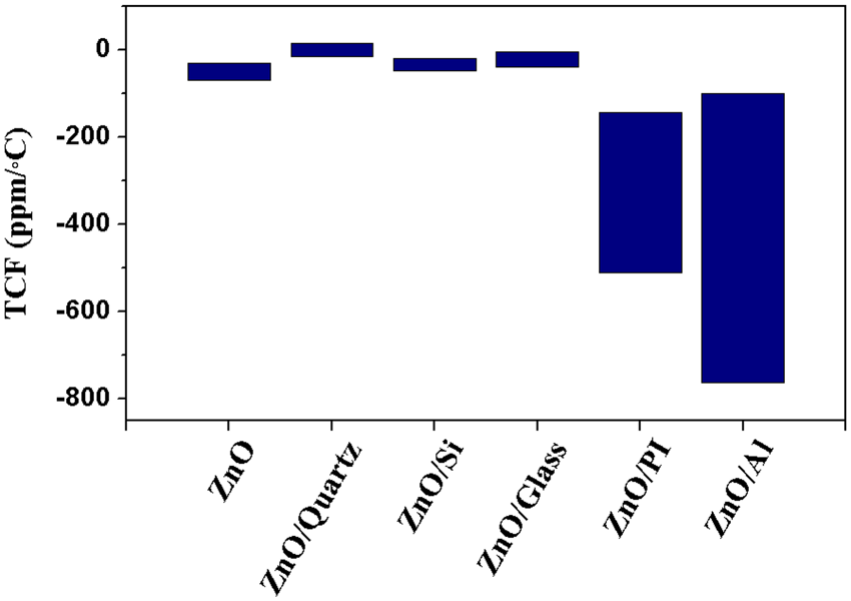
Comparison of TCF readings between ZnO/Al SAWs in this study and ZnO based SAWs on other commonly used substrates reported in literature^16,22,23,25,33–39^.

The data range of all the obtained TCF values of a ZnO coated Al substrate have been summarized in Fig. 5, in which some of the reported TCF values of ZnO film SAW devices fabricated on commonly used substrates are also shown for comparisons. Clearly the ZnO on Al foil offers the highest TCF values reported so far from a literature survey, along with those on polymer substrates. The good performance of the ZnO/Al acoustic wave devices can be explained by the fact that the Al foil has a smaller Young’s modulus^19,32^ and larger TEC and TMC values compared to most other materials commonly used. This could dramatically increase the thermal expansion of the devices during heating/cooling and result in significant variations in the wave velocities with temperature.

ZnO SAW devices prepared on polymer foils and Al foils have great potential for flexible or bendable device applications^24^, which can be applied for flexible temperature sensing applications. According to Fig. 5, ZnO/polymer devices could also have potentially large TCF values (especially at lower resonant frequencies), but the significant attenuation effect in wave propagation (due to the damping nature of soft polymer materials) restricts the fabrication and application of low frequency SAW devices in microfluidics, lab-on-a-chip and actuation applications. ZnO/Al SAW devices, on the other hand, can be easily used for temperature monitoring in lab-on-a-chip applications without reducing their compatibility with multiple functions, such as microfluidics and bio-sampling applications.

## Conclusion

In summary, we have presented an approach to designing layered SAW structures based on ZnO/Al foils and plates to maximize the TCF readings for flexible-substrate temperature sensing applications. Theoretical and FEM simulation were used to optimize the bimorph ZnO/Al structures and achieve high TCF values of the ZnO/Al SAW devices. Example devices were fabricated on ZnO coated Al foil and plates with various thicknesses. A normalized wavelength *λ*/*h* was chosen to describe the changes of the TCF values, and the TCF readings of the ZnO/Al SAW devices show dramatic differences when the normalized wavelength of *λ*/*h* is larger than unity. Results showed that an Al foil (50 μm thick) with 400 μm wavelength achieved the highest TCF reading (−760 ppm/K) ever reported, which is promising for a low cost flexible-substrate temperature sensor applications.

## Methods

### Experimental methods

ZnO films were selected as the piezoelectric layer and were deposited on Al foil (50 ±5 μm), thin (200 μm) and thick (600 μm and 1.6 mm) Al plate using direct-current (DC) magnetron sputtering deposition. During the deposition process, a zinc target with 99.99% purity was used, with an Ar/O_2_ flow ratio of 6/13 sccm, DC target power of 420 W, and a gas pressure of 6 × 10^−4^ mbar. The distance between the target and the sample holder was 100 mm, and the holder was rotated during the deposition to improve the uniformity of thin films. The thicknesses of all ZnO thin films were ~5 μm controlled by the deposition time at a rate of ~5.6 nm/min (Fig. SI1). X-Ray Diffraction (XRD, SIEMENS D5000) was used to obtain the crystalline phases of ZnO thin films and results showed that the film texture is highly c-axis oriented, i.e., with a strong (0002) crystal orientation (see Fig. SI2).

The IDTs composed of 20 nm Cr and 100 nm Au were prepared using a conventional photolithography and lift-off process. The IDTs had wavelength *λ*_0_ values from 64 μm up to 800 μm, with 30 pairs of fingers and an aperture of 5 mm. During the TCF measurement, the temperature was changed from room temperature to around 100 °C within an oven and verified with a temperature sensor fixed on top of the acoustic wave device (more details can be seen from Supplementary Information), and the resonant frequency of the device was recorded using a network analyzer (Keysight HP8753A).

### Data availability

The datasets generated during and/or analysed during the current study are available from the corresponding author on reasonable request.

## Electronic supplementary material

Supplementary Information
