# Supplementary material for: Bimorph material/structure designs for high sensitivity flexible surface acoustic wave temperature sensors
Source: Sci Rep. 2018 Jun 13;8:9052. doi: 10.1038/s41598-018-27324-1 (PMC5998018; doi:10.1038/s41598-018-27324-1)
Supplement: Supplementary file 1 — Supplementary Information [file 41598_2018_27324_MOESM1_ESM.pdf]

Electronic Supplementary Information (ESI)

Bimorph material/structure designs for high sensitivity flexible surface acoustic wave temperature sensors

R Tao,[1,#] S. A. Hasan,[1, #] H. Z. Wang,[2,1,#] J. Zhou,[3] J. T. Luo,[4,1,*] G. McHale,[1] D. Gibson,[5] P. Canyelles-Pericas,[1] M. D. Cooke,[6] D. Wood,[1] Y. Liu,[2] Q. Wu,[1] W.P. Ng,[1] T. Franke,[7] Y. Q. Fu [1,*]

[1] Faculty of Engineering and Environment, Northumbria University, Newcastle upon Tyne, NE1 8ST, UK

[2] State Key Laboratory of Electronic Thin Films and Integrated Devices, University of Electronic Science and Technology of China, Chengdu 610054, People's Republic of China

[3] College of Intelligent Science and Engineering, National University of Defense Technology, Changsha, HuNan, 410073, P. R. China

[4] Shenzhen Key Laboratory of Advanced Thin Films and Applications, College of Physics and Energy, Shenzhen University, Shenzhen, 518060, People's Republic of China

[5] Institute of Thin Films, Sensors & Imaging, University of the West of Scotland, Scottish Universities Physics Alliance, Paisley, PA1 2BE, UK

[6] Department of Engineering, Durham University, South Road, Durham, DH1 3LE, UK

[7] Biomedical Engineering, University of Glasgow, Rankine Building, G12 8LT, Glasgow, UK

#These three authors have equal contributions to this paper.

*Corresponding authors: Prof. J. T. Luo, email: luojt@szu.edu.cn; Prof. Richard Y. Q. Fu, email: richard.fu@northumbria.ac.uk

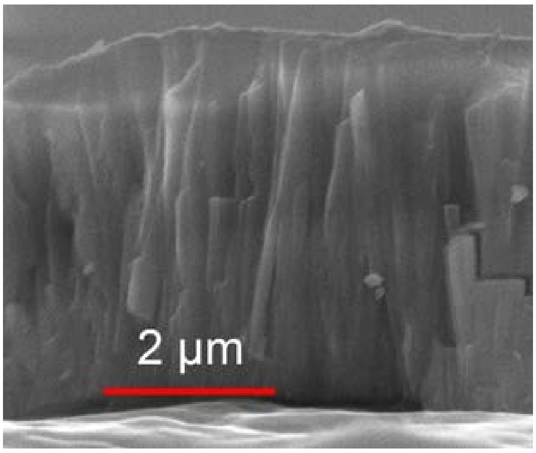

Figure SI1. Cross-section view of ZnO thin films deposited on Al foil.

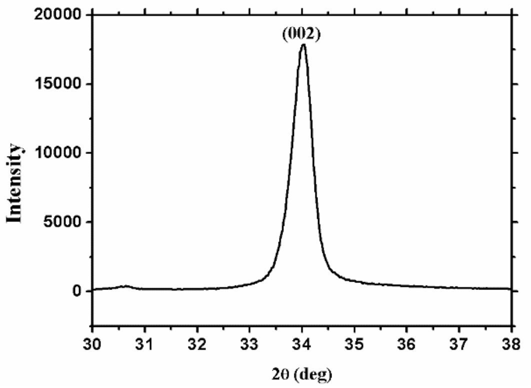

Figure SI2. XRD spectrum of ZnO on Al plate (600 μm thick) indicating the *c*-orientation of ZnO thin film.

(a)

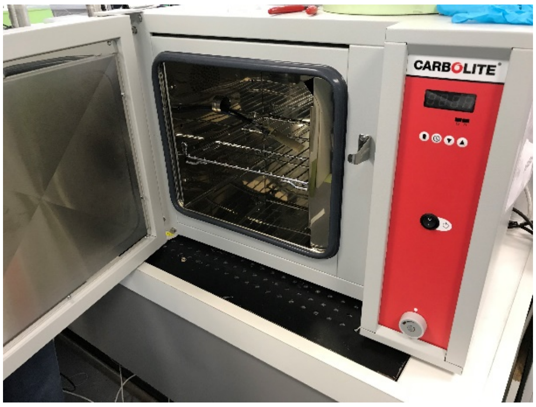

(b)

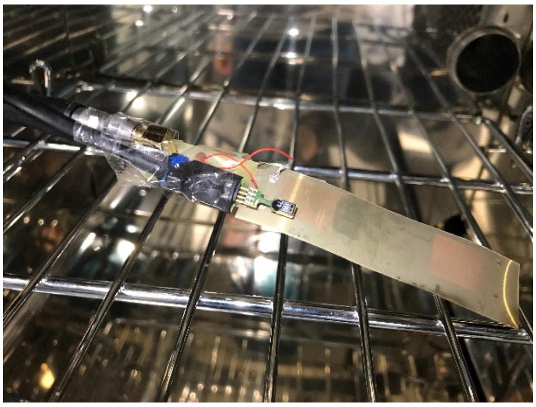

Figure SI3. TCF measurement set-up. (a) Oven chamber where the temperature was changed from room temperature to around 100 ºC. (b) Thermal sensor fixed on top of the acoustic wave device to verify the controlled temperature.
